# Respiratory Symptoms and Urinary Cotinine Levels in Pre-school Children Exposed to Environmental Tobacco Smoke

**DOI:** 10.3389/fpubh.2020.587193

**Published:** 2021-01-26

**Authors:** Yun Wang, Zhiqiang Huang, Dan Luo, Lang Tian, Mi Hu, Shuiyuan Xiao

**Affiliations:** ^1^Department of Social Medicine and Health Management, School of Public Health, Central South University, Hunan, China; ^2^School of Nursing, Anhui College of Traditional Chinese Medical, Anhui, China; ^3^Hunan Academy of Inspection and Quarantine, Hunan, China; ^4^Department of Pediatrics, The Third Xiangya Hospital of Central South University, Hunan, China

**Keywords:** respiratory, urinary cotinine, symptoms, environmental tobacco smoke, preschooler

## Abstract

**Objective:** Preschool children exposed to environmental tobacco smoke (ETS) are at risk of developing adverse respiratory health effects. The present study aimed to investigate the relationship between the effects of cigarette-smoking caregivers on respiratory symptoms and urinary cotinine (UC) levels in children.

**Methods:** A cross-sectional study consisting of 543 children (aged between 5 and 6 years) from 5 kindergartens in central China was conducted using a structured questionnaire. We also analyzed UC levels to investigate the relationship between respiratory symptoms and ETS exposure. We further performed logistic regression analyses to establish the relationship between respiratory symptoms (coughing, rhinorrhea, and sneezing) and UC levels.

**Results:** A total of 71 (13.08%) children had a history of hospital admission with respiratory illnesses including bronchitis and pneumonia over the last 6 months. In addition, 102 (18.78%) children presented coughing symptoms, 114 (20.99%) experienced rhinorrhea and 79 (14.55%) presented sneezing symptoms over the last 6 months. After adjusting the confounders, odds ratio (OR) indicated that the number of cigarettes smoked by a caregiver was associated with coughing (OR = 11.02; 95% CI, 3.72–33.66), rhinorrhea (OR = 41.83; 95% CI, 5.58–313.05) and sneezing (OR = 4.71; 95% CI, 1.33–16.48). Furthermore, UC levels in children with coughing, rhinorrhea and sneezing were significantly higher than in children without respiratory symptoms (*P* = 0.002, *P* < 0.001, *P* < 0.001, respectively).

**Conclusions:** This study reveals that children exposed to ETS have higher levels of UC. Compared with caregiver who non-smoked live with children, smoked cigarettes were highly risk of developing respiratory illnesses in children. Notably, the higher the UC levels the greater the respiratory risk. Our results also signify that UC can be used as an indicator of ETS exposure to inform caregivers of the associated risks, and inform efforts to reduce related effects.

## Introduction

Approximately 40% of children are regularly exposed to environmental tobacco smoke (ETS) resulting in 28% of deaths globally (1). Previous research has demonstrated that exposure to ETS in preschool children causes respiratory illnesses and complications such as bronchitis, pneumonia, upper and lower respiratory tract infections, allergic rhinitis, otitis media, increased risk of asthma, and impaired lung function (2–4). In addition, there are compelling reasons for considering parental smoking behavior as a risk factor for childhood cancers (3, 5). ETS exposure during childhood can contribute to the onset of adult diseases, especially pulmonary and cardiovascular diseases (3).

China constitutes the world's largest population of tobacco consumers, and by 2013, smoking prevalence was higher in men (49.3%) than in women (3.1%) (6). According to the World Health Organization (WHO), approximately 180 million children below 15 years were exposed to ETS daily in 2015 (7). The government of the People's Republic of China instigated a ban on smoking in all public indoor facilities on 14th February, 2011 (8) but this had an insignificant effect on the protection of children against exposure to ETS in homes. Unlike adults, infants and very young children cannot complain, whereas older children affected by ETS may complain but they are often ignored or reprimanded. Furthermore, it is difficult to restrict children from accessing areas of ETS exposure, thus difficult to control inhalation of tobacco smoke. Therefore, children are more vulnerable to ETS exposure and the resulting negative outcomes. Studies conducted in the recent past have revealed that ETS exposure and oxidative stress are strongly associated with numerous diseases (9–12).

Previous studies have also reported a positive correlation between ETS exposure and the cotinine level in children (13–16). However, a few studies have been conducted on the relationship between ETS exposure, UC levels and respiratory symptoms in a population of children aged 5–6 years. Therefore, the current cross-sectional study aimed to: [1] determine the relationship between smoking caregivers and respiratory symptoms in children aged 5-6 years, [2] describe the relationship between respiratory symptoms and UC levels in children aged between 5 and 6 years, and [3] analyze the association between UC levels and respiratory symptoms such as coughing, rhinorrhea and sneezing in children exposed to ETS.

## Subjects and Methods

### Study Population

A cross-sectional study using a two-stage, simple random sampling was performed between August and October 2013 in Changsha (central China). The study population included 543 preschool children aged 5–6 years randomly drawn from all classes in the five kindergartens. We used the following inclusion criteria: children aged 5–6 years; a self-reported smoking condition of caregiver who lived with a child, individuals who do not use coal for cooking and heating at home, and participants not restricted to give information. All participants in the present study signed written informed consent forms, and participating children had the consent of their parents or caregivers. The study protocol was approved by the Ethics Committee of the Clinical Pharmacology Research, Institute of Central South University (Changsha, China; approval ref: CTXY-12023). A total of 543 children, aged between 5 and 6 years and their caregivers were enrolled for the study. In this study, a caregiver was defined as a close person who lives and takes care of the child at home. The project was initially introduced to caregivers of children in the 5 kindergartens before it was implemented. The cross-sectional study analyzed data on ETS exposure at 5-6 years, respiratory symptoms and urine samples of participating children.

### Sociodemographic Variables

The sociodemographic variables included the following: age of caregivers (≤35 years old = 0; >35 years old = 1); sex of caregivers (male = 1; female = 2); house size (≤60 m^2^ = 0; >60 m^2^ = 1); caregiver educational level (≤12 years = 1; >12 years = 2); occupation type (part-time job = 1; full-time job = 2); and family monthly income (≤4,499 Yuan = 1; >4,500 Yuan = 2; 4,499 Yuan approximately US$ 643). Total scores of education, occupation and family income values were calculated as socio-economic status (SES) stratification, with high scores indicating improved SES. In this study, high SES was defined as a total of five or more and low SES as a total of less than five. The proportion of caregivers in the high and low SES groups was 59.5% and 40.5%, respectively.

### ETS Exposure and Respiratory Symptoms in Children

A caregiver completed a structural questionnaire, which had previously indicated 100% response rate and good validation (17) to collect information about their smoking behavior. This information was collected from everyone living with a child and included smoking (yes/no), and the number of cigarettes smoked at home. Smoking behavior such as “daily cigarette consumption” was assigned a categorical variable and coded in the binary regression analysis. The coding system used was as follows; 0 cigarettes/day = 0, 1−10 cigarettes/day = 1, 11–20 cigarettes/day = 2, and >20 cigarettes/day = 3. Respiratory symptoms were traced by asking the caregivers questions on the occurrence of cough, wheezing, production of sputum, sore throat, running nose and hoarseness. Respiratory diseases including asthma, bronchitis, pneumonia and allergic rhinitis were designated as doctor-diagnosed diseases over the last 12 months. Moreover, we collected additional information on the respiratory health of the children by asking questions such as “what do you think of your child's health in the recent three months?” The responses included: [1] Very good (never ill, no cough, no running nose, no doctor or hospital visits); [2] Good [occasionally ill (1–2 times)]; [3] Bad [frequently ill (3–6 times)]; [4] Very bad (always ill). Caregivers were asked the following questions to collect information on respiratory symptoms: if their child has had any respiratory symptoms in the last 6 months and dichotomous responses (yes/no) were used to confirm the symptoms. “Does your child usually have a sore or itchy throat or any other throat discomfort?,” “Does your child usually have a cough in the morning after waking up?,” “Does your child experience frequent rhinorrhea or running nose?,” and “Has your child been diagnosed with the following respiratory illnesses, [1] bronchitis (yes/no) and [2] pneumonia (yes/no) during the last 12 months?”

### Determination of Urinary Cotinine Levels Based on ETS Exposure

Urinary samples were collected, coded and frozen at −20°C for further analyses. We used an analytical method for determining UC levels in children exposed to ETS based on a stable isotope dilution by gas chromatography–tandem mass spectrometry (GC-MS/MS) as previously described (18, 19), and applied a quantification limit (LOQ) of 0.1 ng/ml. Urinary samples were extracted and purified with chloroform. The internal standard (cotinine-d_3_) was added to urine samples and centrifuged at 7,000 × g for 10 min at room temperature. Extracts were determined by GC–MS/MS in multiple reaction monitoring mode.

### Statistical Analyses

Statistical analyses were conducted using SPSS software version 22.0 (IBM Corp, New York, NY, USA). The population characteristics and data on exposure to ETS were presented as percentages. Univariate logistic regression was used to assess the association between the number of cigarettes smoked by a caregiver per day and variables associated with the respiratory symptoms including coughing, rhinorrhea and sneezing due to ETS exposure. Data on UC were presented as median and interquartile range (IQR) because of non-normal distribution. The age of children was presented as means ± standard deviations (mean ± SD). The Mann–Whitney *U*-test was used to analyze UC levels between two groups; for example, differences in SES and respiratory symptoms and illnesses, as well as UC and exposure or non-exposure to ETS. The original UC levels were used as continuous variables in multivariate logistic regression analysis at 0.05 and 0.10 significance levels for exclusion from models. The Kruskal–Wallis test was used to determine statistically significant differences in the numbers of cigarettes smoked by the caregivers, and Nemenyi test (median test) performed in cases where the data had a skewed distribution. The following variables were included in the multivariate logistic regression model: age of caregiver, sex, education, family income, occupation, house size, cigarettes smoked and the UC level. Odds ratios (ORs) were calculated at 95% confidence intervals (CI) for cigarettes smoked and respiratory symptoms at *p* < 0.05.

## Results

The response rate for all questionnaires used in the present study was 100%. A total of 321 (59.1%) of the 543 children enrolled in the study were boys, with an average age of 5.5 ± 0.6 years (Table 1). We observed that exposure of children to ETS increased with the number of cigarettes smoked by their caregivers. The UC level of ETS exposure in children who lived with caregivers who smoked 1-10 cigarettes per day was 2.29 ng/mL (IQR: 1.90, 2.71), caregivers who smoked 11-20 cigarettes per day was 4.15 ng/mL (IQR: 3.01, 5.85) and for caregivers who smoked more than 20 cigarettes per day 7.73 ng/mL (IQR: 4.15, 15.46). Compared with no smoker at home, significant differences of UC level in children were observed in the group that caregiver smoked 1-10 cigarettes per day (χ^2^= 195.393, *p* < 0.001). Moreover, significant differences of UC level in children were observed between caregiver smoked 11-20 cigarettes smokers in the household (χ^2^ = 186.771, *p* < 0.001) and more than 20 cigarettes smoker groups in the household (χ^2^ = 152.600, *p* < 0.001) (Table 1). Children from disadvantaged households constituted 60% of the children enrolled for the study, and their mean UC levels were higher than those of children from advantaged households. Significant differences were observed in UC levels between children from disadvantaged and advantaged households (χ^2^= −2.305, *p* = 0.021) (Table 1).

**Table 1:** Caregiver/child characteristics and urinary cotinine levels (ng/mL).

**Demographic**	**% Total sample (n)**	**Median (Interquartile Range) (ng/mL)**	**χ^2^**	***p***
**Child's age, years**				
5.5 ± 0.6 (mean ± SD)	543	2.70 (1.76–4.76)		
Child's sex			−0.016	0.987
Boy	59.1 (321)	2.67 (1.73–4.77)		
Girl	40.9 (222)	2.71 (1.79–4.77)		
Caregiver's age, years			−0.390	0.697
≤ 35	65.4 (355)	2.86 (1.34–5.07)		
>35	34.6 (188)	2.59 (1.85–4.37)		
Education			−1.742	0.081
≤ 12 years	42.9 (233)	3.14 (1.60–5.49)		
>12 years	57.1 (310)	3.63 (1.79–4.36)		
Occupation			−0.760	0.447
Full-time job	72.2 (392)	2.62 (1.79–4.77)		
Part-time job	27.8 (151)	2.92 (3.40–4.35)		
Monthly household income (RMB)^*^			−1.663	0.096
≤ 4499	30.4 (165)	3.23 (1.68–5.98)		
≥4500	69.6 (378)	2.59 (1.78–4.43)		
House size (m^2^)			−7.340	<0.001
≤ 60	7.4 (40)	0.63 (0.46–1.68)		
>60	92.6 (503)	2.91 (1.84–5.02)		
SES^#^ scores			−2.305	0.021
≤ 5	59.5 (323)	3.01(1.75–5.37)		
>5	40.5 (220)	2.35 (1.76–4.16)		
No. of caregiver smokers			289.397	<0.001
0	118	0.72 (0.47–0.91)		
1	310	2.97 (2.12–5.43)		
>1	115	4.46 (3.10–7.31)		
Smoked cigarettes/day			362.462Δ	<0.001
(1) 0	21.7 (118)	0.72 (0.47–0.91)	(1)-(2) 195.393	<0.001
(2) 1–10	29.8 (162)	2.29 (1.90–2.71)	(1)-(3) 186.771	<0.001
(3) 11–20	33.0 (179)	4.15 (3.01–5.85)	(1)-(4) 152.600	<0.001
(4) >20	15.5 (84)	7.73 (4.15-15.46)	(2)-(3) 114.275	<0.001
			(2)-(4) 93.714	<0.001
			(3)-(4) 21.379	<0.001

* *RMB, renminbi (Chinese currency)*;

# *SES, socio-economic status*.

Δ *Kruskal-Wallis test*.

We analyzed respiratory symptoms, illnesses and urinary cotinine levels (ng/ml) of children (Table 2). Coughing was recorded in 102 children; rhinorrhea was recorded in 114 children and sneezing was recorded in 79 children. Significant differences were observed in coughing [2.96 ng/mL (2.29–5.04) *vs*. 2.65 ng/mL (1.46–4.75), *p* = 0.002], rhinorrhea [3.64 ng/mL (2.43–9.03) *vs*. 2.43 ng/mL (1.32–4.47), *p* < 0.001] and sneezing [4.44 ng/mL (2.70–6.18) *vs*. 2.50 ng/mL (1.57–4.47)*, p* < 0.001], respectively. And the UC levels of children with coughing, rhinorrhea and sneezing symptoms were higher than those without the symptoms. The numbers of children diagnosed with bronchitis and pneumonia in the last 12 months were 110 and 67, respectively. Furthermore, UC levels of children exhibited a significant difference, and the UC levels of children diagnosed with bronchitis and pneumonia were higher than in children without these conditions (*p* < 0.001). UC levels in children with bronchitis were higher than in children without bronchitis [3.06 ng/mL (2.15–5.31) *vs*. 2.53 ng/mL (1.46–4.59)], and the difference was significant (*p* < 0.001). UC levels in children with pneumonia were also higher than in children without pneumonia [4.34 ng/mL (2.71–6.86) *vs*. 2.51 ng/mL (1.67–4.52)], and the difference was significant (*p* < 0.001). Approximately 24.3% (*n* = 132) of the surveyed children had no respiratory illnesses whereas 75.7% (*n* = 411) reported a history of respiratory illness in the last 6 months, and the difference in their UC levels was significant (*p* < 0.001). Children with more incidences of respiratory infections in the last 6 months had higher UC levels than those without previous respiratory infections (*p* < 0.001). Notably, 86.9% (*n* = 472) of the surveyed children had not been hospitalized with respiratory illnesses whereas 13.1% (*n* = 71) reported a history of hospitalization with respiratory illnesses in the last 6 months, their UC levels were significantly different (*p* < 0.001). Children with higher UC levels were more likely to be hospitalized than those with lower UC levels in the past 6 months. In addition, significant differences in the UC levels were observed with phlegm [3.67 ng/mL (2.90–5.29) *vs*. 2.65 ng/mL (1.73–4.75), *p* = 0.018], otitis media [4.42 ng/mL (2.86–9.09) *vs*. 2.68 ng/mL (1.75–4.76), *p* = 0.031] and allergic sinusitis [4.97 ng/mL (2.69–9.15) *vs*. 2.68 ng/mL (1.73–4.74), *p* = 0.011]. No significant differences were observed in UC levels of children with throat discomfort, asthma and wheezing.

**Table 2:** Respiratory symptoms, illness and urinary cotinine levels (ng/ml) of children.

**Respiratory symptoms and illness**		***n***	**UCMedian (Interquartile range) (ng/mL)**	***Z^**2**^***	***p***
Throat discomfort	No	269	2.69 (1.80–4.77)	−0.708	0.479
	Yes	274	2.74 (1.70–4.77)		
Cough	No	441	2.65 (1.46–4.75)	−3.146	0.002
	Yes	102	2.96 (2.29–5.04)		
Cough in three months in the year	No	530	2.69 (1.73–4.75)	−1.605	0.108
	Yes	13	3.81 (2.15–9.44)		
Phlegm in the morning	No	525	2.65 (1.73–4.75)	−2.365	0.018
	Yes	18	3.67 (2.90–5.29)		
wheezing	No	541	4.10 (3.75–4.44)	−0.090	0.928
	Yes	2	2.85 (/)		
Rhinorrhea	No	429	2.43 (1.32–4.47)	−5.698	<0.001
	Yes	114	3.64 (2.43–9.03)		
Allergic sinusitis	No	531	2.68 (1.73–4.74)	−2.536	0.011
	Yes	12	4.97 (2.69–9.15)		
Asthma	No	531	2.68 (1.73–4.76)	−1.962	0.050
	Yes	12	3.56 (2.72–13.54)		
Sneeze	No	464	2.50 (1.57–4.47)	−4.855	<0.001
	Yes	79	4.44 (2.70–6.18)		
Bronchitis	No	433	2.53 (1.46–4.59)	−3.545	<0.001
	Yes	110	3.06 (2.15–5.31)		
Pneumonia	No	476	2.51 (1.67–4.52)	−5.023	<0.001
	Yes	67	4.34 (2.71–6.86)		
Otitis media	No	531	2.68 (1.75–4.76)	−2.161	0.031
	Yes	11	4.42 (2.86–9.09)		
Respiratory infections in the past 6 months	No	132	1.26 (0.71–3.13)	−7.302	<0.001
	Yes	411	3.01 (1.96–5.23)		
Respiratory illness admitted to a hospital in the past 6 months	No	472	2.47 (1.68–4.52)	−4.735	<0.001
	Yes	71	4.03 (2.85–6.82)		

*UC, urinary cotinine*.

The results of univariate analysis of respiratory symptoms and illnesses in children, and the number of cigarettes smoked daily by the caregiver indicated a crude association in OR between the number of cigarettes smoked daily by a caregiver and coughing, rhinorrhea, sneezing, bronchitis and pneumonia (Table 3). Caregivers who smoked 1–10 cigarettes (OR = 12.358, 95% CI: 4.317–35.383), 11–20 cigarettes (OR = 6.683, 95% CI: 2.304–19.379) and more than 20 cigarettes (OR = 6.196, 95% CI: 1.976–19.425) per day were associated with increased risk of cough in children compared to non-smoking caregivers lived with children. Moreover, a similar trend was observed between smoked by a caregiver and rhinorrhea, sneezing, bronchitis and pneumonia. Multivariate analysis of the respiratory symptoms and illnesses, and the number of cigarettes smoked daily by a caregiver indicated an association in adjusted odds ratios (AOR) between the number of cigarettes smoked daily by a caregiver and cough, rhinorrhea and sneezing, with variations in age of caregiver, sex, education, occupation, monthly family income, house size, UC levels of children (as continuous variables) and numbers of smoked cigarettes (Table 4). Analysis of the corresponding coughing symptoms indicated an AOR of 11.020 (CI 3.718–32.664). Compared to caregivers who were non-smokers to those who smoked living with children, our results also indicated increased risk estimates for smoking 11–20 (AOR 5.878, CI 1.913–18.058) and more than 20 (AOR 5.364, CI: 1.532–18.775) cigarettes per day. Compared to caregivers who were non-smokers to those who smoking living with children, analysis of rhinorrhea revealed significantly increased risk estimates for total cigarettes smoked per day (*P* < 0.001). Similarly, analysis of sneezing further revealed that risk estimates increased. Sneezing indicated an AOR of 4.706 (CI: 1.344–16.477). An increase in risk estimates was observed in the group smoking 11–20 (AOR 8.234, CI: 2.365–28.676) cigarettes and more than 20 (AOR 6.286, CI: 1.583–24.972) cigarettes per day. We also observed a significant association between elevated risk estimates for sneezing and the number of cigarettes smoked per day (*p* < 0.001). This apparent elevation in risk estimates was observed between smoking, bronchitis and pneumonia. Caregivers who smoked 1-10 cigarettes per day had a higher risk estimate for bronchitis in children than in non-smoking caregivers (AOR 4.933, CI: 1.913–12.718). This positive correlation was also observed between smoking caregivers and pneumonia in children (AOR 3.872, CI: 1.008–14.876). The trends for respiratory symptoms and illnesses based on smoking habits (socio-demographic characteristics, numbers of smoked cigarettes and UC level adjustment) are presented in Table 4.

**Table 3:** Odds ratio (OR) and 95% confident interval (95% CI) describing children's respiratory symptoms and illness and caregiver daily smoked cigarettes.

**Variable**		**Cough**			**Rhinorrhea**			**Sneeze**			**Bronchitis**			**Pneumonia**		
	***N***	**(*n*)**	**OR**	**95% (CI)^#^**	**(*n*)**	**OR**	**95% (CI)^#^**	**(*n*)**	**OR**	**95% (CI)^#^**	**(*n*)**	**OR**	**95% (CI)^#^**	**(*n*)**	**OR**	**95% (CI)^#^**
No smoking	118	4	1.0		1	1.0		3	1.0		6	1.0		3	1.0	
1–10 cigarettes/day	162	49	12.358	4.317–35.383	51	53.757	7.304–395.646	22	6.024	1.759–20.634	34	4.958	2.008–12.247	14	3.626	1.018–12.919
11–20 cigarettes/day	179	34	6.683	2.304–19.379	44	38.133	5.174–281.069	39	10.679	3.217–35.452	49	7.036	2.905–17.041	35	9.317	2.794–31.067
>20 cigarettes/day	84	15	6.196	1.976–19.425	18	31.909	4.165–244.457	15	8.333	2.329–29.823	21	6.222	2.386–16.224	15	8.333	2.329–29.823

*OR, Odds ratio*;

# *95% CI, 95% confident interval*.

**Table 4:** Adjusted odds ratio (AOR) and 95% confident interval (95%CI) describing children's respiratory symptoms and illness and caregiver daily smoked cigarettes.

**Variable**		**Cough**			**Rhinorrhea**			**Sneeze**			**Bronchitis**			**Pneumonia**		
	***N***	**(*n*)**	**AOR**	**95% (CI)^#^**	**(*n*)**	**AOR**	**95% (CI)^#^**	**(*n*)**	**AOR**	**95% (CI)^#^**	**(*n*)**	**AOR**	**95% (CI)^#^**	**(*n*)**	**AOR**	**95% (CI)^#^**
No smoking	118	4	1.0		1	1.0		3	1.0		6	1.0		3	1.0	
1–10 cigarettes/day	162	49	11.020	3.718–32.664	51	41.834	5.581–313.054	22	4.706	1.344–16.477	34	4.933	1.913–12.718	14	3.872	1.008–14.876
11–20 cigarettes/day	179	34	5.878	1.913–18.058	44	21.592	2.837–164.347	39	8.234	2.365–28.676	49	6.452	2.505–16.618	35	9.560	2.635–34.694
>20 cigarettes/day	84	15	5.364	1.532–18.775	18	13.916	1.690–114.609	15	6.286	1.583–24.972	21	5.653	1.928–16.581	15	7.947	1.908–33.098

# *Adjusted for caregiver's age, sex, education, occupation, monthly family income, house size, children's urinary cotinine levels, and caregiver smoked cigarettes*.

In the present study, significant differences were observed between SES and UC levels, and it was evident that children from disadvantaged households had higher UC levels than those from advantaged families (Table 5). A comparison of average UC levels with respiratory symptoms and illnesses in children based on SES scores revealed an increasing UC trend in children from disadvantaged households. The SES scores and UC levels of children with respiratory symptoms and illnesses revealed a strong negative correlation. Furthermore, significant differences were recorded in the respiratory symptoms and illnesses with UC levels in advantaged (SES>5) and disadvantaged households (SES ≤ 5). The geometric mean values of UC concentrations in children with coughing symptoms from disadvantaged and advantaged households were 3.97 ng/mL (2.62–5.22) and 2.46 ng/mL (1.99–4.41), respectively, and the difference was significant (*Z* = −2.358, *p* = 0.018). A significant difference was observed in rhinorrhea [4.86 ng/mL (2.71–9.93) *vs*.2.98 ng/mL (2.28–4.50), *p* = 0.013), sneezing [4.80 ng/mL (3.55–6.86) *vs*. 2.95 ng/mL (2.09–5.16), *p* = 0.007], bronchitis [3.81 ng/mL (2.68–6.48) *vs*. 2.42 ng/mL (2.03–4.15), *p* = 0.013], pneumonia [4.73 ng/mL (3.66–8.06) *vs*. 3.31 ng/mL (2.35–5.30), *p* = 0.006], respectively. There was a significantly difference in UC concentrations of children between disadvantaged and advantaged households (*p* < 0.05).

**Table 5:** Social economic status, urinary cotinine levels, respiratory symptoms, and illness of children.

**Social economic status (*N*)**	**Cough**		**Rhinorrhea**		**Sneeze**		**Bronchitis**		**Pneumonia**	
	**(*n*)**	**UC median (interquartile range) (ng/mL)**	**(n)**	**UC median (interquartile range) (ng/mL)**	**(n)**	**UC median (interquartile range) (ng/mL)**	**(n)**	**UC median (interquartile range) (ng/mL)**	**(n)**	**UC median (interquartile range) (ng/mL)**
≤ 5 (323)	53	3.97 (2.62–5.22)	58	4.86 (2.71–9.93)	43	4.80 (3.55–6.86)	67	3.81 (2.68–6.48)	40	4.73 (3.66–8.06)
>5 (220)	49	2.46 (1.99–4.41)	56	2.98 (2.28–4.50)	36	2.95 (2.09–5.16)	43	2.42 (2.03–4.15)	27	3.31 (2.35–5.30)
*Z*		−2.358		−2.488		−2.687		−2.490		−2.767
*P*		0.018		0.013		0.007		0.013		0.006

*UC, urinary cotinine*.

## Discussion

In the current study, we obtained satisfactory response from caregivers that were a representative sample of the target population through the questionnaires. The investigation had a large sample size which significantly strengthens our study. Our results indicated that exposure of children aged between 5 and 6 years to ETS largely occurs in homes with smoking caregivers, and this exposure decreases with increase in the social class. We observed a positive correlation between increased cigarette smoking by the caregivers and occurrence of respiratory symptoms, and UC levels in children. Remarkably, the prevalence of ETS exposure in children at home is 78.3%, which differs across different countries. For example, the prevalence of ETS exposure in children was reported to be 21.6% in Spain (20), 37.9% in the U.S. (21), 63% in Israel (22), and 46.3% in Ghana (23). These reports indicate that exposure to ETS in children aged 5–6 years as seen in this study was high.

Although the use of questionnaires to assess ETS exposure in children has limited validity, the inclusion of UC which is a reliable indicator of short-term ETS exposure in children guarantees validity of the results (24). Findings of the present study also demonstrated that smoking by caregivers results in higher UC levels in children, consequently causing respiratory symptoms and illnesses. Based on the potential effects of ETS exposure on the respiratory tract, the median concentrations of UC levels in most children with respiratory symptoms and illnesses significantly differed from those of children without respiratory symptoms and illnesses. Moreover, we established that high UC levels were considerably associated with higher risk of coughing, phlegm, allergic sinusitis, rhinorrhea and sneezing, bronchitis and pneumonia in children. For example, the median UC values for children without cough symptoms was 2.65 ng/mL and children with cough symptoms was 2.96 ng/mL. The mean UC level in children with cough symptoms was 1.1 times higher than that of children without cough symptoms. Previous studies have reported that cough reflex is impaired in seemingly healthy children living with smokers (25). Generally, coughing, phlegm and sneezing are vital responses that help in clearing chemicals and particulate irritants including those in tobacco smoke from the airways. However, children aged 5-6 years old who are affected by ETS cannot complain, and may be ignored or reprimanded if they complain. Irreversible impaired respiratory tract clearance may persist, thus increasing susceptibility to respiratory illnesses. Furthermore, there was a significant association between higher UC levels, bronchitis and pneumonia. The UC levels of children with bronchitis and pneumonia were 1.2 and 1.7 respectively times higher than those of children without bronchitis and pneumonia. These results suggested that children diagnosed with bronchitis and pneumonia had higher UC levels, which translated to higher probability of hospitalization. Several studies have demonstrated the relationship between ETS exposure and risk of bronchitis and pneumonia (26, 27). In addition, exposed to environmental tobacco smoke children with respiratory symptoms and illness can be caused by multiple challenges such as the burdens of a caregiver, absence from work, economic cost and decreased health-related quality of life in children (28–30). We did not perform further analysis on the risk estimates of children with allergic sinusitis, otitis media and a dose–response association to the daily smoking by caregivers because of the small positive sample size. Nevertheless, a few studies have reported a positive correlation between otitis media and smoking caregivers (31, 32). A large sample size using a longitudinal design in a future study is necessary to confirm our findings.

Univariate analysis indicated an increased risk between smoking caregivers and respiratory symptoms and illnesses in children. Cigarette smoking was a risk factor for children with coughs, rhinorrhea, sneezing, bronchitis and pneumonia in comparison with non-smoking caregivers. Moreover, multivariate analysis revealed significant associations in the odds ratio association between cigarette smoking caregivers and coughing, rhinorrhea and sneezing, adjusted variables for caregiver's age, sex, education, occupation, monthly family income, house size, UC level in children (as continuous variables), and number of smoked cigarettes. The adverse effects of cigarette smoke from caregivers and ETS on the respiratory health outcomes of a child are plausible. In this study, the numbers of smoked cigarettes were highly associated with high risk estimates of coughing, rhinorrhea and sneezing in children. Compared with caregiver who non-smoked lived with children, smoking 1-10 cigarettes per day, was associated with 11.0-fold increase in the odds ratio for having coughs, 41.8-fold increase for having rhinorrhea, and 4.7-fold increase for sneezing. This finding is consistent with the findings of previous studies (33, 34). In the current study, the odds ratio for ETS exposure in children was higher than in children who were not exposed to ETS in the case of non-smoking caregivers. Strikingly, we observed a positive correlation between smoking cigarettes and respiratory symptoms, although the increase was not linear. There were reasons as following: Firstly, consideration of social expectations for smoking behavior, the smoker maybe underreported. Secondly, since it is a cross-sectional study, the causal sequence of time is not clear. However, compared to caregivers who were non-smokers to those who smoked living with children, these results signified that smoking cigarette increased the risk estimate ratios for respiratory symptoms and illnesses in children. This can be partially attributed to the uniqueness of a child, which is influenced by a caregiver. Additionally, the reason may be the small sample size of positive cases thus a larger sample is needed to validate these findings. Nevertheless, results of the present study provide sufficient evidence that exposure of children to tobacco smoke from caregivers increases the risk of respiratory symptoms.

We also established that ETS exposure decreases with increase in the socioeconomic status of a family. Notably, high UC levels were significantly associated with children living in households with low socio-economic status. The measure of socio-economic status used in the study was a proxy measure based on education, occupation and family income monthly. Numerous studies have reported the relationship between socio-economic status and childhood ETS exposure. Low levels of parental education have been identified as a significant contributor to ETS exposure in children (35, 36).

The detection biomarker of based on laboratory approach, there are added value of taking children and caregiver smoking history. On the one hand, self-reports have the advantage of low cost and ease of administration, but raise questions of reliability and validity. So, using of multiple measures of exposure to environmental tobacco smoke which may lead respondents to believe that their reports are verifiable. On the other hand, based on the level of urinary cotinine, it helps us to evaluate the dose of exposure accurately and the relationship between exposure and disease.

The present study revealed a relationship between quantifiable UC with respiratory symptoms and illnesses in children exposed to cigarette smoke at home. However, this study had several limitations. Firstly, we adopted a cross-sectional approach, therefore general conclusions on the temporal order or causal relationships cannot be drawn. Secondly, all the investigated variables were self-reported thus recall bias may exist. Therefore, it will be imperative to collect objective data such as urine samples from the children to validate the findings of this study experimentally, and more detailed information on the socio-economic status of families considering the significance of our results. Thirdly, there was no clinical confirmation of bronchitis and pneumonia through health facility records, thus respondents might have over-reported and under-reported symptoms of these diseases.

Exposure assessment is a necessary and vital part of environmental epidemiology studies and intervention studies. The detection of urine cotinine in children as a way was applied to screen, surveil and evaluate the effect of intervention. There are some suggestions in the future study. Firstly, a longitudinal randomized control trial is a better study design. Secondly, thirdhand smoke mixes pose a potential health hazard to children. Future intervention studies need to focus on full protection for children across all settings. Thirdly, collecting the data from doctor's record and diagnosis of children help to measure accurately the relationship between exposure and disease.

A large number of children are currently at risk or will be at risk of the adverse effects associated with unhealthy indoor air quality caused by ETS exposure. The smoking habit of caregivers increases risk ratios of respiratory symptoms such as coughing, rhinorrhea, sneezing in children. UC levels in children with respiratory symptoms and illnesses increase when they are exposed to ETS. The quantitative values indicating the effects of children exposure to ETS and smoking caregivers can be used to educate caregivers and as a potential deterrent factor to stop smoking, and should be considered in families with children exposed to ETS. We recommend highlighting the risks of ETS exposure in children as an intervention strategy, targeting caregivers who smoke at home. A formal trial of this intervention based on the approach that targets caregivers who help children should be implemented.

## Data Availability Statement

The raw data supporting the conclusions of this article will be made available by the authors, without undue reservation.

## Ethics Statement

The studies involving human participants were reviewed and approved by The Ethics Committee of the Clinical Pharmacology Research, Institute of Central South University (Changsha, China; approval ref: CTXY-12023). Written informed consent to participate in this study was provided by the participants' legal guardian/next of kin.

## Author Contributions

Conceptualization, SX. Data curation, YW and SX. Formal analysis, YW, ZH, and DL. Funding acquisition, YW and SX. Investigation, YW, LT, and MH. Methodology, ZH, DL, and MH. Project administration, SX. Resources, LT. Writing—original draft, YW and SX. All authors contributed to the article and approved the submitted version.

## Conflict of Interest

The authors declare that the research was conducted in the absence of any commercial or financial relationships that could be construed as a potential conflict of interest.
